# Ferroelectric translational antiphase boundaries in nonpolar materials

**DOI:** 10.1038/ncomms4031

**Published:** 2014-01-08

**Authors:** Xian-Kui Wei, Alexander K. Tagantsev, Alexander Kvasov, Krystian Roleder, Chun-Lin Jia, Nava Setter

**Affiliations:** 1 Ceramics Laboratory, Swiss Federal Institute of Technology Lausanne (EPFL), Lausanne CH-1015, Switzerland; 2 Peter Grünberg Institute and Ernst Ruska Center for Microscopy and Spectroscopy with Electrons, Research Center Jülich, 52425 Jülich, Germany; 3 Institute of Physics, University of Silesia, Katowice 40007, Poland; 4 International Centre of Dielectric Research, The School of Electronic and Information Engineering, Xi'an Jiaotong University, Xi'an 710049, China

## Abstract

Ferroelectric materials are heavily used in electro-mechanics and electronics. Inside the ferroelectric, domain walls separate regions in which the spontaneous polarization is differently oriented. Properties of ferroelectric domain walls can differ from those of the domains themselves, leading to new exploitable phenomena. Even more exciting is that a non-ferroelectric material may have domain boundaries that are ferroelectric. Many materials possess translational antiphase boundaries. Such boundaries could be interesting entities to carry information if they were ferroelectric. Here we show first that antiphase boundaries in antiferroelectrics may possess ferroelectricity. We then identify these boundaries in the classical antiferroelectric lead zirconate and evidence their polarity by electron microscopy using negative spherical-aberration imaging technique. *Ab initio* modelling confirms the polar bi-stable nature of the walls. Ferroelectric antiphase boundaries could make high-density non-volatile memory; in comparison with the magnetic domain wall memory, they do not require current for operation and are an order of magnitude thinner.

## Abstract (web summary)

In ferroelectrics, the domain walls can have different properties than the domains themselves. Here, Wei *et al.* show that certain domain walls in antiferroelectric materials are ferroelectric, which makes them interesting candidates for new non-volatile memory concepts.

## Graphical abstract

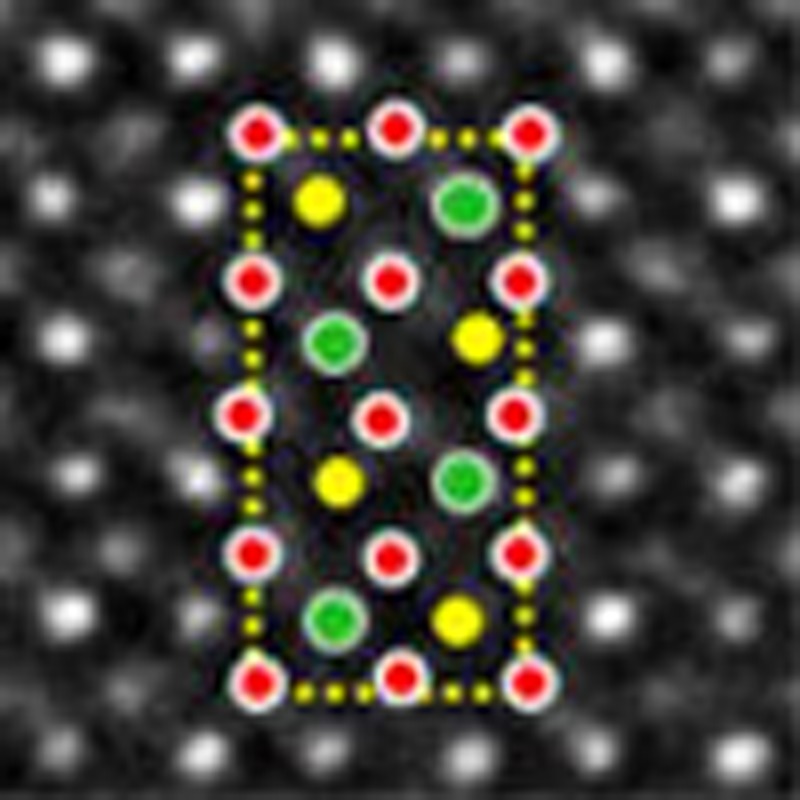

Domain boundaries make an intriguing and challenging research subject because of their peculiar properties and promising perspective in designing nanoelectronic devices. One typical paradigm is the discovery of electronic conductivity at ferroelectric domain walls in multiferroic oxides such as BiFeO_3_ (refs 1, 2, 3) and ErMnO_3_ (ref. 4). Charged domain walls in the ubiquitous ferroelectric BaTiO_3_ showed electron-gas-like conductivity while the individual domains remained excellent insulators^5^. Large photovoltages were generated by domain walls, attractive for photovoltaic devices^6^. These properties are particularly attractive because domain walls can be created, annihilated, rewritten and displaced electrically inside the material, potentially leading to agile nanoelectronics.

Recently, the polarity of twin boundaries in centrosymmetric CaTiO_3_ was shown^7^. Possible polarity of domain boundaries in nonpolar materials and, to a greater extent, local ferroelectricity in such boundaries make these objects attractive for both fundamental science and possible practical future applications.

How to identify materials with ferroelectric boundaries? Here based on a Landau theory treatment, we argue that antiphase boundaries in antiferroelectrics are likely to be ferroelectric. Implementing this result, we perform an atomic-scale study of the prototypical antiferroelectric lead zirconate PbZrO_3_ (PZ), using negative spherical-aberration imaging (NCSI) technique^8^ in an aberration-corrected transmission electron microscope (TEM) and demonstrate that antiphase boundaries with a π phase shift of the order parameter exhibit polarity, which, in view of the symmetry of the system, implies the existence of local ferroelectricity.

## Results

### Ferroelectricity in antiferroelectric domain walls

Antiferroelectrics constitute a large group of dielectric materials that can be experimentally recognized by a structural phase transition between two nonpolar phases with a strong dielectric anomaly at the high temperature side of the transition^9^,^10^,^11^. Antiferroelectricity is a result of the interruption of an imminent ferroelectric phase transition having Curie temperature of *T*_0_ by a structural phase transition^12^ at a slightly higher temperature *T*_A_. This interruption occurs because of repulsive interaction between polarization and a structural order parameter appearing at *T*_A_.

A simple two instabilities Landau-type theory^12^,^13^,^14^,^15^ rationalizes the antiferroelectric behaviour using the following free energy expansion in terms of polarization, *P*, and structural order parameter, *ξ*,


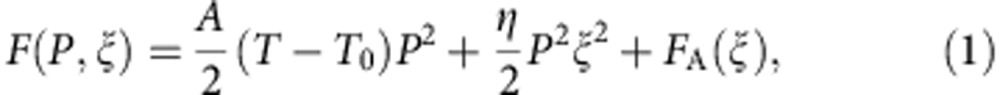


A>0, and *T*_0_ is the Curie-Weiss temperature of the dielectric anomaly (see Supplementary Note 1 for more details on the two-instability theory of antiferroelectricity). Here the coefficient *η>*0 controls the repulsive biquadratic coupling between polarization and structural order parameter. The transition at *T*_A_ is described by the contribution to the free energy *F*_A_(*ξ*), implying softening of a lattice mode associated with the order parameter *ξ*. For the low-temperature phase where the order parameter of the transition, *ξ*, acquires a spontaneous value of *ξ*_0_, the susceptibility, defined as 
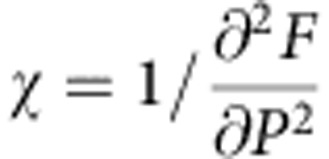
, takes the form


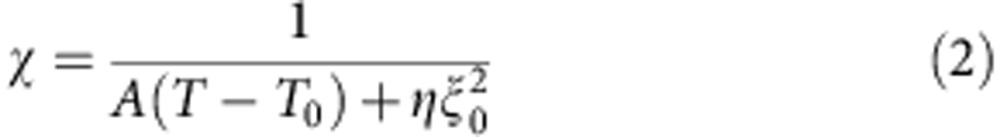


Equation (2) corresponds to antiferroelectric-type anomaly if 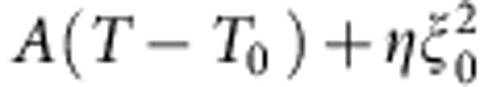 increases on cooling. This is possible if the increase in *ξ*_0_ with lowering temperature dominates the behaviour of this term, which can be assured by large enough coupling constant *η*.

The above scenario suggests that domain boundaries provide favourable conditions for the development of local ferroelectricity. This can be elucidated considering the simplest type of ferroic domain boundary, the so-called Ising wall. In such wall, the order parameter *ξ* passes through zero in the middle of the boundary. Thus, in the middle of the boundary, the suppressing effect of the structural ordering (with respect to the *ξ* parameter) on the ferroelectric instability vanishes, creating favourable conditions for the development of local ferroelectricity at *T<T*_0_.

### Antiphase boundaries in PZ as candidates for ferroelectricity

**Figure 1:**
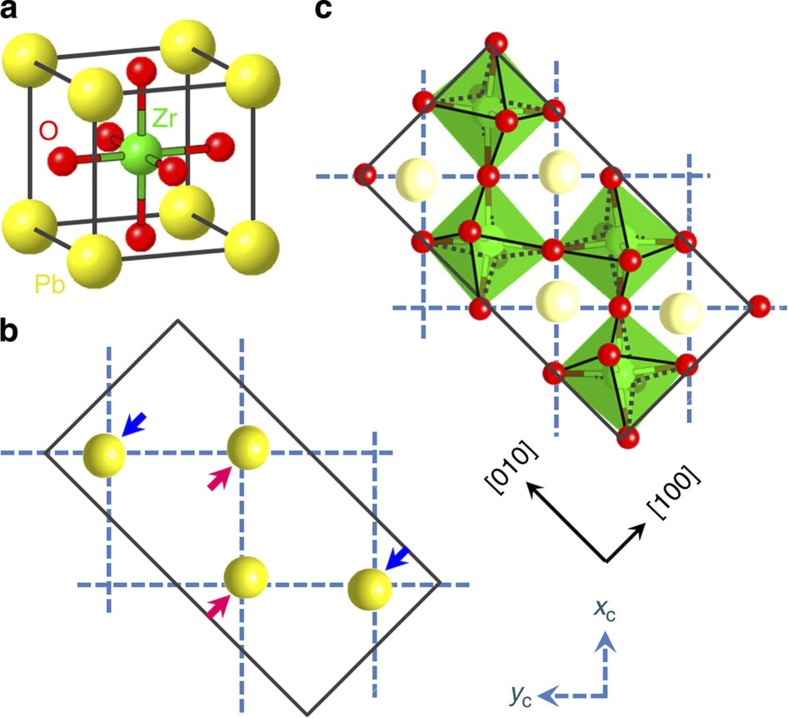
Structure of lead zirconate. (**a**) The cubic unit cell. Lattice modes relevant to the phase transition into the orthorhombic phase. (**b**) Lead displacements in the 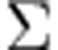 mode. (**c**) Oxygen-octahedron rotations in the R-mode. In **b**,**c**, the projections of the orthorhombic unit cells onto the *ab* plane (rectangles) are shown. The crystallographic axes of the orthorhombic phase ([100] and [010]) and of the pseudocubic phase (*x*_*c*_ and *y*_*c*_) are shown.

The concept presented above can be applied to PZ. However, in PZ the situation is more complicated. At high temperatures, PZ has the ideal cubic perovskite structure shown in Fig. 1a. After cooling through a first-order phase transition at *T*_A_~500 K, the structure changes from cubic 
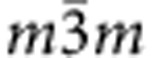
 to orthorhombic *mmm*. The structural changes at the transition can be presented as a combination of displacements in two lattice modes^16^, one corresponding to the 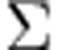 point (the wave vector 
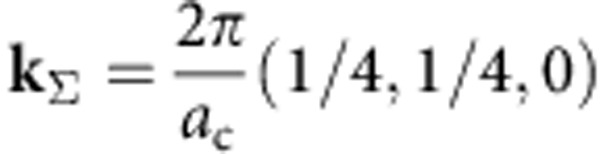
; *a*_c_ is the lattice constant of the high-temperature cubic phase) in the Brillouin zone and the other corresponding to the *R* point (
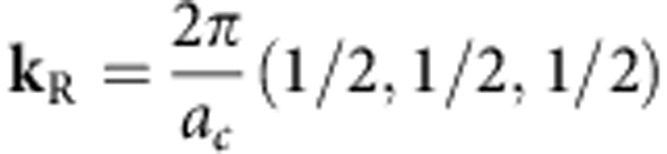
). The distortions associated with the 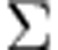 point are mainly related to displacements of lead ions (Fig. 1b), while those associated with the *R* point derive from antiphase rotations of the oxygen octahedra (Fig. 1c) about the crystallographic axes of the cubic phase. Thus, the transition is governed by a mixed order parameter, containing 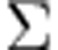-point and *R*-point-related components.

The possibility of local ferroelectricity in domain boundaries can be discussed referring to the 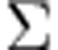-point-related component of the order parameter, corresponding to the so-called 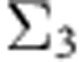 mode of the parent cubic structure, with lead displacements having the form^12^,^17^


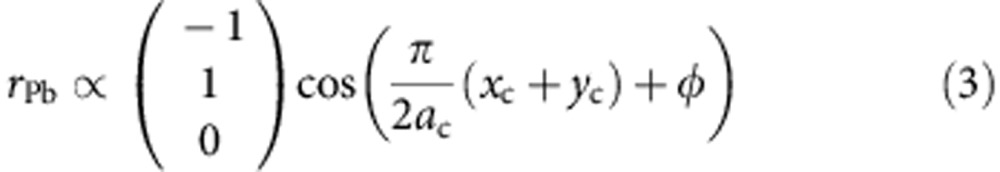


where *x*_c_=*n*_1_*a*_c_, *y*_c_=*n*_2_*a*_c_ (*n*_1_ and *n*_2_ are integers) are the coordinates in the cubic lattice frame and *φ* is the phase of the modulation.

**Figure 2:**
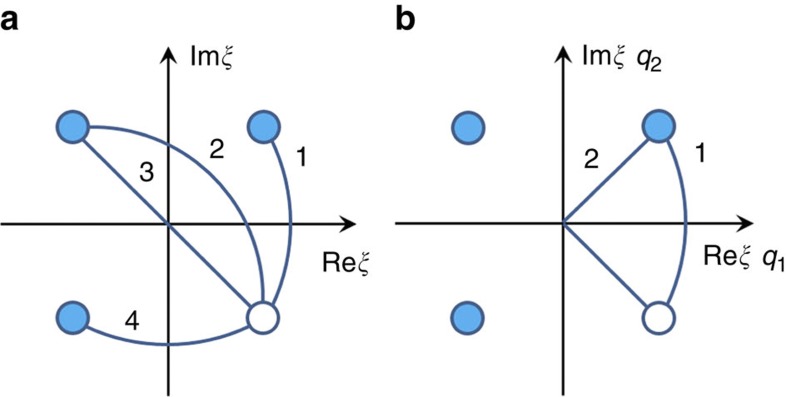
Mapping of the translational domain states and boundaries. Mapping of the translational domain states and boundaries inside a single orientational domain of PZ on the plane of the complex order parameter. Circles—translational domain states. Lines—translational boundaries. The boundaries linking the domain state marked by empty circle with the other states are shown with numbered lines. (**a**) Naturally expected translational boundaries.The phase shifts Δ*φ* of the modulation of the lead displacements in the walls are: line 1—the *π*/2 wall, Δ*φ*=*π*/2; lines 2 and 3—the *π* walls (APB), Δ*φ*=*π*; line 4—the 3/2*π* wall, Δ*φ*=3*π*/2. In the π-wall (line ‘3’) the order parameter passes through zero. In view of the above discussion, in such wall the suppressing effect of the order parameter on the ferroelectric instability is minimal, making the π-wall the most favourable for the occurrence of local ferroelectricity. (**b**) Mappings of possible *π*/2 walls on the plane (Re *ξ*, Im *ξ*) of the complex amplitude of the order parameter in PZ—lines 1 and 2. Mappings of possible ferroelectric walls on the plane (*q*_1_, *q*_2_) of the two-component order parameter in GMO—lines 1 and 2.

The condensation of the order parameter linked to this mode is associated with a fourfold increase in the number of atoms per unit cell as schematically depicted in Fig. 1b. As a result, two types of domain states form: orientational and translational^18^. The orientational states (ferroelastic twins) differ by the orientation of the atomic displacements. The translational domain states that correspond to a given orientational state can be turned from one to another by shifting the lattice by *a*_c_, 2*a*_c_ or 3*a*_c_, corresponding to a fraction of a lattice translation vector of the low-temperature phase. In view of the quadrupling of the unit cell, there are four translational domain states for each orientational state. In terms of Equation (3), lead displacements in these states correspond to *φ*=*π*/4, 3*π*/4, 5*π*/4 and 7*π*/4 (ref. 12). These states can be visualized in the plane of the complex order parameter *ξ*=*ρe*^*iφ*^, corresponding to the points marked with four circles (Fig. 2a), where *φ* is the phase of the order parameter and *ρ* is its modulus, proportional to the magnitude of lead displacements.

Three types of translational boundaries, corresponding to phase shifts of *π*/2, *π* and 3*π/*2 can separate the four translational states. The possible mappings of these translational boundaries are schematically shown in Fig. 2a with solid lines. Mapping ‘3’ is of a particular interest for the appearance of the local ferroelectricity. It corresponds to a π-wall (so-called antiphase boundary (APB))—that is, having a phase shift of *π*, at the middle of which the order parameter passes through the zero point hence the suppressing effect of the order parameter on the ferroelectric instability is reduced. Following this argument one might contemplate a *π*/2 translational domain boundary where the order parameter passes as well through zero (mapping ‘2’ in Fig. 2b) and wonder whether also in this case the ferroelectric instability is favoured. No theory of translational domain boundaries in PZ is available at present to quantitatively address this problem. However, such a problem has been addressed for an improper ferroelectric by Fouskova and Fousek^19^. These authors compared the energies of two variants of a domain wall in gadolinium molibdate (GMO), which have mapping on the plane of the two-component order parameter (*q*1, *q*2) marked with lines ‘1’ and ‘2’ in Fig. 2b. They demonstrated that the variant corresponding to mapping ‘2’, which passes through the origin is energetically unfavourable compared with the rotational variant, corresponding to mapping ‘1’. The problem in GMO is isomorphous to that in PZ to within the substitution (Re *ξ*, Im *ξ*) for (*q*1, *q*2). The results obtained for GMO enable us to speculate that passing through zero of the order parameter in a *π*/2 translational domain boundary in PZ is quite improbable. On the same lines, we argue that in orientational (twin) domain boundaries, which are also comparable to the walls treated by Fousek and Fouskova^19^, the order parameter is not expected to pass through zero in the wall neither. This implies that there is no special reason for the appearance of local ferroelectricity in twin boundaries, although such event cannot be excluded. It is therefore the *π* wall that is promising for the local ferroelectricity. We propose that APBs in PZ—that is, translational boundaries with phase shifts of π—are good candidates for the occurrence of local ferroelectricity.

### The thickness of APBs in ferroics

The above discussion motivates experimental search for ferroelectricity in APBs in PZ. For such research, it seems appropriate to specify the notion APB in ferroics, which is not fully identical to that commonly used in non-ferroic materials.

By definition, any two elementary unit cells in a perfect crystalline structure can be superimposed by translation with an elementary vector belonging to the set of the lattice translation vectors of the structure. Some crystals exhibit distinct neighbouring regions, where a unit cell in one can superimpose a unit cell in the other by the application of a vector differing from an allowed lattice translation vector by approximately half of an elementary lattice translation vector. In this case, the boundary separating the two regions is called APB. Hereafter, the aforementioned translation vector is called an APB vector. We term this primary definition of the APB *definition-0*.

**Figure 3:**
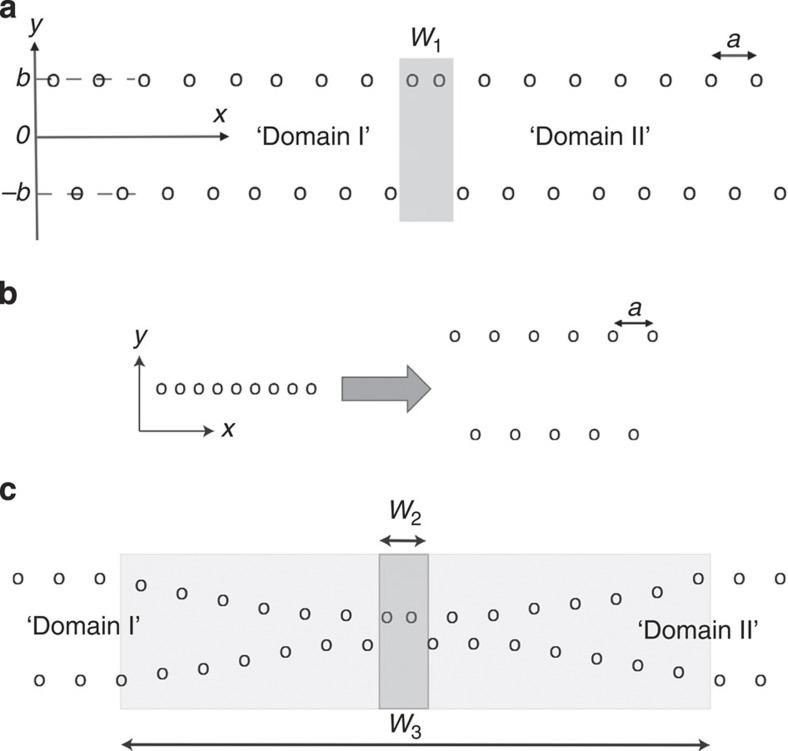
APB in a ‘non-ferroic’ versus APB in a ferroic. (**a**) A 2D schematic of an APB in a non-ferroic. Here only one chain of atoms parallel to the *Ox* direction is shown and the whole 2D ‘crystal’ can be obtained by repeating this chain in the *Oy* direction with a period *c*. Regions ‘domain I’ and ‘domain II’ have both a perfect crystalline structure with period *a*. Yet, in order to superpose elementary cells of region I on region II, a vector is needed, which differs from a translational vectors by *a*/2. This vector does not belong to the set of elementary lattice translation vectors of the structure, all equal *na*, *n* being integer numbers. The antiphase boundary *w*_1_ is marked grey and its width is about *a*/2. (**b**) 2D schematic of a structural phase transition in a ferroic. In each phase, only one chain of atoms is shown; the whole 2D ‘crystal’ can be obtained by repeating the chain in the perpendicular direction with the period *c*. At the transition, the period in the direction of the chain changes from *a*/2 to *a*. (**c**) Schematic of an APB in the 2D ferroic introduced in Fig. 2b. The thickness of the APB consistent with its primary definition (*definition-0*) is *w*_3_. According to *definition-1*, one would attribute to the APB the thickness *w*_2_.

Figure 3a shows a two-dimensional (2D) schematic of an APB. Here only one chain of atoms parallel to the *Ox* direction is shown and the whole 2D ‘crystal’ can be obtained by repeating the chain in the *Oy* direction with a period *c*. In this drawing, the APB vector differs from a lattice translation vector exactly by *a*/2—that is, by a half of the elementary translation vector. In a more realistic model, the separation between the neighbouring ‘atoms’ in the *Ox* directions should be *a*/2 only inside regions of ‘domain I’ and ‘domain II’ of the structure, while at the APB it should evidently be slightly different from this value.

For the model depicted in Fig. 3b, another definition of the APB (termed hereafter *definition-1*) can be introduced based on *definition-0* given above. Specifically, it is seen that the regions ‘I’ and ‘II’ can be characterized by the alternation of the signs of the atomic displacements along the *Oy* direction, which are either *b* or −*b*. As is clear from Fig. 3a, one can identify the presence of the APB by a shift of the phase of this alienation and ascribe the position of the APB to the place where this phase shift takes place. For the model depicted in Fig. 3a, the APB can be viewed as a layer that is *a*/2 thick in the *Ox* direction. In terms of *definition-0*, by removing away this layer, the periodicity throughout the whole system, which was violated by the presence of the APB, is restored. In terms of *definition-1*, at this layer, the regular alternation of the atomic displacements in the *Ox* direction is violated. On the basis of any of the two definitions, we can attribute to the APB the width of *a* and speak about its location with the accuracy of the interatomic distance. *Definition-1* is more convenient in practical work compared with *definition-0*.

The model depicted in Fig. 3a corresponds to the situation in non-ferroic crystals. Meanwhile, for ferroics, that situation is, in general, different and requires a special treatment. The new features are as follows: (i) *definition-1* is not consistent any more with the primary *definition-0*, and (ii) a thickness equal to the correlation length rather than the interatomic distance should be ascribed to the APB. This situation is discussed next.

In ferroics, APBs occur once a structural phase transition is accompanied with a unit cell multiplication. Figure 3b shows the evolution of the structure at such a phase transition in a ferroic ‘material’ where the unit cell volume is doubled at the transition and the period in the *Ox* direction changes from *a*/2 to *a*. Specifically, the structural evolution shown in this figure corresponds to the case, where, in the ordered phase, the structure is in a single domain state. It is seen that such single domain structure is equivalent to that inside regions ‘I’ and ‘II’ of the non-ferroic 2D crystal shown in Fig. 3a.

In such a ferroic 2D crystal, APBs may readily occur, separating between so-called translational domains. Specifically, for a ferroic having a second-order phase transition or of a first order close to the second-order transition (the typical situation that enables the Landau theory treatment), in an APB, the structure evolves gradually between the two translational domains on a spatial scale of about the correlation length *ζ.* The primary statement of the Landau theory is that *ζ* is much larger than the lattice constant of the material. In practice, because of an interplay of numerical factors such difference may not be very large; however, conceptually, *ζ* should be larger than the lattice constant. A ferroic ‘material’ exhibiting two translational domains marked with ‘I’ and ‘II’, which are separated by an APB, is schematically depicted in Fig. 3c.

We now pose the question of the thickness and position of a ferroic APB. Using *definition-0*, one treats the APB as the area different from the translational invariant domains ‘I’ and ‘II’. Note that the translational invariance requires not only the periodicity in the *Ox* direction but also the identity of the atomic displacements in the *Oy* direction. Thus, we consider the APB as an object having the thickness *w*_3_≅*ζ*. The APB is the whole region, with the thickness of about *ζ*, where the lattice periodicity is violated. Once the APB contains many distorted unit cells, one can say that, in an APB, the modulus of the order parameter deviates from its bulk value.

Can one apply *definition-1* to an APB in ferroics? In *definition-1*, we considered the APB to be atomically thin (in Fig. 3c it is *w*_2_) and specified its location to within the interatomic distance. However, in ferroics this definition loses its essential feature. Specifically, in non-ferroics *definition-1* is equivalent to the primary *definition-0*. On the contrary, in ferroics, *definition-1* is in conflict with the primary *definition-0*. In a ferroic material, two unit cells separated just by the APB of *definition-1* cannot be superimposed by any translational vector at all; thus, it is impossible to speak about a phase shift at it. Thus, strictly speaking, when applied to ferroics, *definition-1* of the APB does not correspond to the term ‘APB’ itself.

To summarize, APBs in ferroics are objects of a finite volume, not just a stepwise break of the periodicity.

### Electron microscopy

Following the above analysis, we investigated PZ to search for local polarity in its domain boundaries. Employing the NCSI technique^8^ in an aberration-corrected TEM combined with quantum mechanical and optical simulation, we imaged and measured the atomic positions, including those of oxygen.

**Figure 4:**
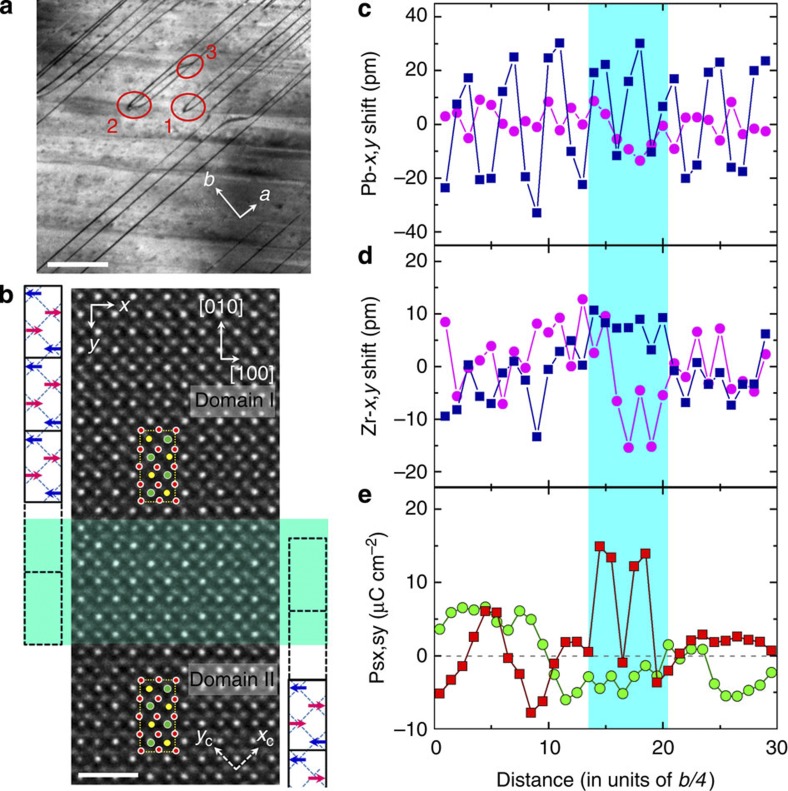
Morphology and atomic details of translational boundaries in the antiferroelectric PZ crystal. (**a**) Dark-field image with superposed orthorhombic axes shows morphology of the translational boundaries (dark lines). Topological features of these boundaries are marked by the red circles: ‘1’—annihilation of two APBs, ‘2’—annihilation of one APB and two *π*/2 walls and ‘3’—split of an APB into two *π*/2 walls. Scale bar, 200 nm. (**b**) Atomic-resolution image of an APB between two translational domains recorded under NCSI conditions with the incident electron beam parallel to the [001] direction. In two domains, two identically defined orthorhombic unit cells are highlighted. Lead atom displacements denoted by blue and pink arrows are represented in the schematic orthorhombic blocks to the left (domain I) and the right (domain II) of the image. By shifting the orthorhombic cell, the APB (the shaded cyan area) can be evidenced by the conflict of half a unit cell in between these two domains. Scale bar, 1 nm. (**c**,**d**) Displacements of Pb and Zr atoms with respect to the atomic positions of the cubic phase, averaged over the planes along the *x* direction as function of the plane positions along the *y* direction. Blue squares: in-plane (the *x* direction, [100] in Fig. 1) displacements parallel to the wall; pink circles: out-of-plane (the *y* direction, [010] in Fig. 1) displacements normal to the wall. (**e**) Dipole moment density obtained by averaging the dipole moments of a ‘sliding’ orthorhombic unit cell plotted as a function of the centres of the sliding cells. Red squares: in-plane polarity; green circles: out-of-plane polarity.

Figure 4a is a dark-field image of a PZ crystal, showing translational domains separated by domain walls (dark line contrast). These translational domains correspond to a single orientational domain. The topological nature of the domain walls allows reactions between them, provided the conservation of the total phase shift (topological charge) is kept. Encircled and marked ‘1’, ‘2’ and ‘3’ are annihilation of two APBs, annihilation of one APB and two *π*/2 walls and split of an APB into two *π/*2 walls, respectively.

A high-resolution TEM image of an APB is shown in Fig. 4b. It was recorded under NCSI condition with the electron beam along the crystallographic [001] direction. One readily checks that the two highlighted unit cells exhibit a *π* shift in the phase of the order parameter. The identity of these two unit cells can be checked with lead displacements. Passing a pair of rows in the [010] direction, the sign of lead displacements alternates as shown with the blue/pink arrows in Fig. 4b, following the law of the alienation dictated by Equation (3). The number of lead-containing rows between the two highlighted unit cells is 10 (and not a multiple of 4), evidencing the *π* phase shift and the existence of the APB region. On the basis of image simulation, the atomic positions corresponding to the experimental image are obtained from the model structure.

A violation of the correlation in the antipolar in-plane Pb displacements is clearly seen in the APB area (Fig. 4c). Owing to the depolarizing effect, polarity is expected only in the boundary plane—that is, along the [100] orthorhombic axis. Scrutinizing the *x*-atomic displacements shown in Fig. 4d, we find a systematic unipolar displacements of Zr in 7 elementary cubes (pseudocubic unit cells) with an average value of about 8 pm. This hints to a dipole moment inside the wall, having a dipole moment density of about 11 μC cm^−2^, obtained taking *a*_*c*_=0.413 nm and six electron charges for the Zr Born charge (see Supplementary Note 2). Details about oxygen displacements are provided in Methods. Re-arrangement of the oxygen atomic positions at the APB area clearly reveals breaking of the antiphase-correlated rotation of the octahedra. Within the accuracy of the experiments, width of the APB can be evaluated as one to two orthorhombic unit cells along the [010] direction.

To evaluate the polarity of the APB we used the following approach: we calculated the dipole moments based on the displacements of all the ions from their positions in the cubic phase associated with their Born charges. Then, we averaged these moments over the projection of the orthorhombic unit cell on the *ab* plane using a ‘sliding’ unit cell (see Methods). Figure 4e shows the dipole moment density obtained this way, as a function of the centre of the sliding unit cells. Note that this figure shows a deviation from zero polarity in the centrosymmetric domain bulk. This deviation indicates the measurement errors (s.d. *σ*<5 μC cm^−2^) of the polarization calculated this way. This relatively low accuracy is not surprizing when calculating the dipole moments of a 40-atom unit cell of the orthorhombic PZ. Importantly, one can ascribe in-plane polarity to the APB area, corresponding to some 14 μC cm^−2^, definitely exceeding the ‘noise’ level. The contribution of B-site to the polarity in the wall can be taken as an indication that the B-site atoms contribute to the ferroelectric soft mode of the material. From Fig. 4e, it is also clear that no polarity normal to the boundary can be ascribed to APB, which is consistent with the expected manifestation of the depolarizing effect.

### Simulations

**Figure 5:**
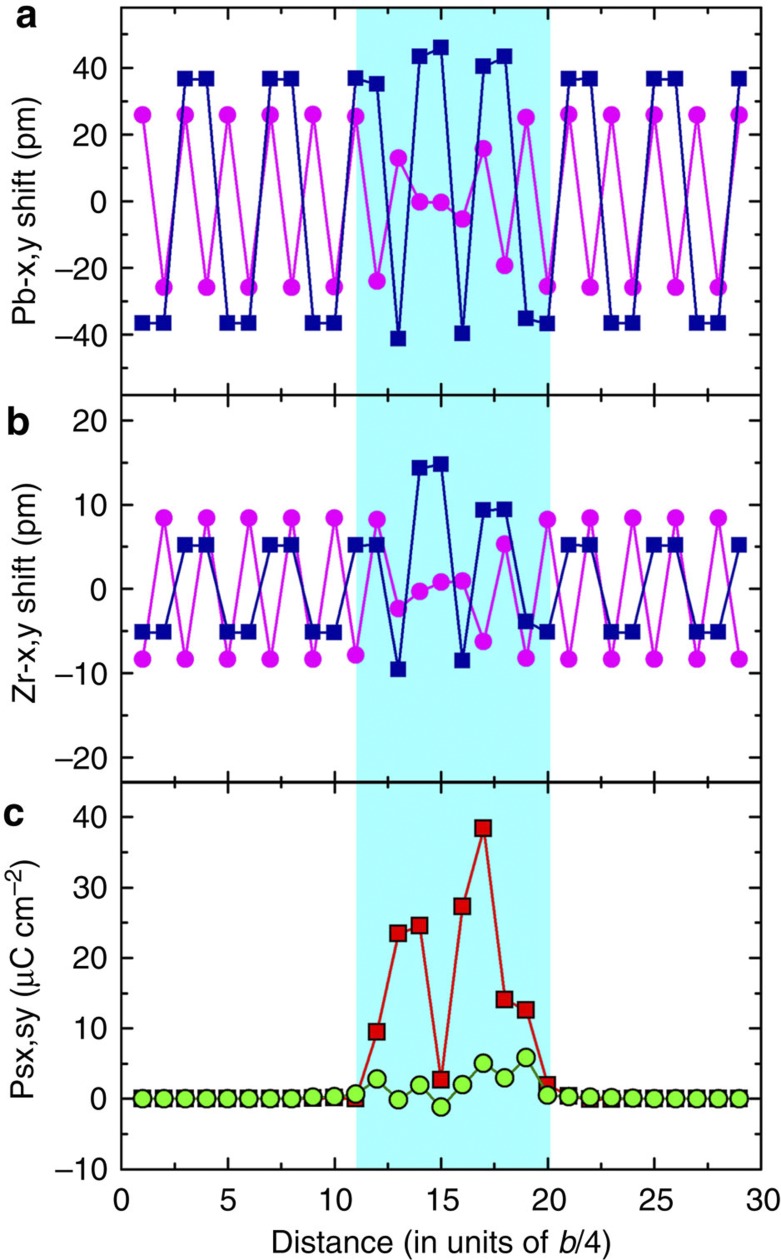
Results of *ab initio* calculations. (**a**,**b**) Displacements of Pb and Zr atoms with respect to the atomic positions of the cubic structure, squares: in-plane (*x* direction) displacements parallel to the wall, circles: out-of-plane (*y* direction) displacements normal to the wall. (**c**) Dipole moment density for the ‘sliding’ orthorhombic unit cell plotted as a function of the centres of the sliding cells; squares: in-plane polarity, circles: out-of-plane polarity.

The presence of local polarity in the APB in PZ was also investigated by first principles calculations (Supplementary Note 2). The results confirmed the observed polarity and agreed qualitatively with the experimental results. The corresponding atom displacements and polarity are shown in Fig. 5. Using *ab initio* results we have estimated the surface formation energy of the considered here *π*-wall as 190 mJ m^−2^. Our simulations also demonstate the bi-stability of the polar state of APBs in PZ.

## Discussion

Thus, the electron microscopy results provide experimental evidence for the anticipated polarity in APBs in the centrosymmetric PZ and the *ab initio* calculations give a further support to the experimental observations. As the observed polarity is the result of spontaneous symmetry breaking, the phenomenon observed at the APB is a signature of local ferroelectricity. This is supported by first principle calculations that reveals the bi-stability of the polarity at the APB. Although the switchability of the polarity was not demonstrated experimentally, the moderate values of the spontaneous polarization found in the APB suggest moderate values of its thermodynamic coercive field, which behaves as the cube of spontaneous polarization^18^, promising that the polarity is not only bi-stable but also switchable.

It is worth pointing out the qualitative difference between the phenomenon reported here and a scenario for magnetism in a domain boundary in an antiferromagnet. Roughly speaking, the antiferromagnetic ordering is a result of negative exchange interaction between the spins of the system. In antiferromagnetic walls, the exchange interaction may change the sign leading to a local ferromagnetic ordering, the order made by the same spins that are interacting in an antiferromagnetic way in the domains themselves. In our case, the ‘antipolar’ displacements inside the domains are mainly associated with the A-site (Pb) ions, whereas the polarity in the wall is driven by all the atoms: the A-site (Pb), B-site (Zr) atom and oxygens. This is consistent with the two-mode scenario for antiferroelectricity and is different from the mechanisms controlling antiferromagnetism.

The polar antiphase domain boundaries reported above are expected to be bi-stable and mobile. The bi-stability of polar domain walls in a nonpolar material is readily expected in view of the symmetry arguments. Understanding the mobility of the APBs is less evident. Since translational domains are identical in terms of all materials tensors no macroscopic stimulus can lift the energy degeneracy of the domains. However, under electric field, **E**, the dipole moment density in the wall is associated with the energy density −**PE**. This implies force acting on the wall once the electric field is inhomogeneous,—for example, when electric field gradient is applied across the wall.

Ferroelectric antiphase domain boundaries in antiferroelectrics can be viewed as functional elements, 1–10 nm wide, which carry information. Unlike twin domain walls, the polar translational walls are non-ferroelastic, which makes them strain-free, and thus even more appealing for potential information-carrying elements. In comparison with the attractive magnetic domain wall memory^20^, they do not require current for operation and are an order of magnitude thinner, thus adding potentially a new element for future high-density information storage.

## Methods

### Material preparation

Lead–zirconate crystals were grown by flux method. The PbO–B_2_O_3_ mixture (soaking at 1,050 K) was used as a solvent. The temperature of the melt was reduced at a rate 2 K h^−1^ down to 850 K. The remaining melt decanted and attached to the crucible walls were cooled to room temperature at a rate of 10 K h^−1^. Then, the crystals were etched in dilute acetic acid to remove residues of the solidified flux.

### Imaging experiments

The TEM specimens were prepared using focused ion beam system. To remove the contamination and the damaged layers, plasma cleaning and NanoMill Model 1040 system operated at 500 V were used to clean and mill the lamella samples. The lamella samples were heated above *T*_A_ and then cooled at ≈9 K min^−1^ to recover the orthorhombic phase at the thin edges. The TEM investigation was performed on an FEI Titan 80–300 microscope with a *C*_s_ corrector for the objective lens. The available point resolution was better than 0.08 nm at an operating voltage of 300 kV. The experimental image was filtered to minimize the effect of contrast noise. Structure modelling and image simulation were carried out using the CrystalKit-MacTempas software package.

**Figure 6:**
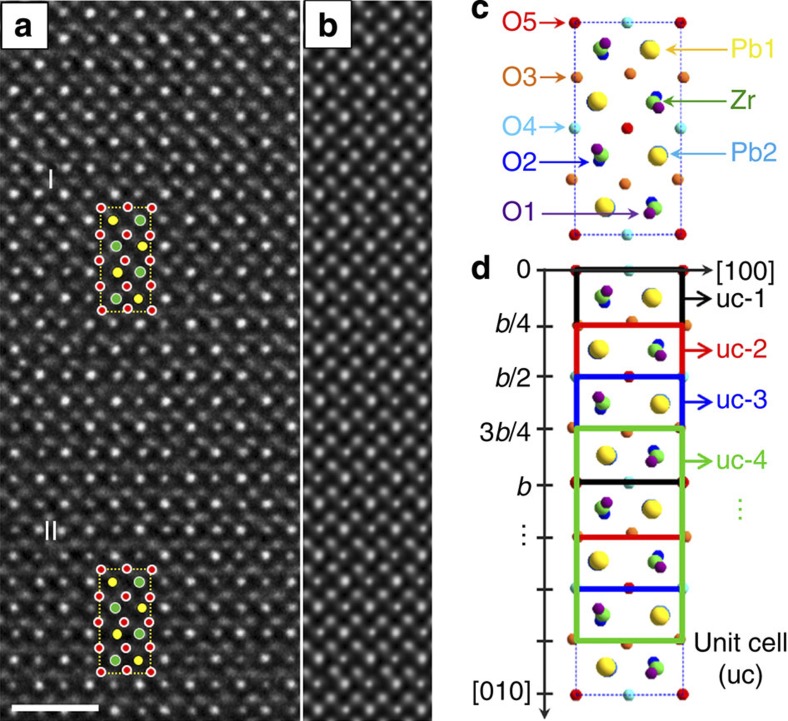
TEM results. Comparison of the filtered experimental image (**a**) with the simulated image (**b**). Scale bar, 1 nm. The simulation parameters: sample thickness=9.87 nm, defocus=6.2 nm, twofold astigmatism=2 nm, threefold astigmatism=50 nm, coma=80 nm, crystal tilt of 0.8 mrad and vibration of 0.04 nm. (**c**) The orthorhombic unit cell of PZ projected on (001) plane showing the crystallographic sites of all atoms. (**d**) Schematic diagram of the ‘sliding’ orthorhombic unit cell. On the basis of the structure model derived from the image simulation, the spontaneous polarization is calculated for each unit cell, then the cell is shifted by *b*/4.

The experimental image filtered using a special method^21^ was quantified in the following way. First, a least-square fit was performed by 2D Gaussian profiles to each of the individual intensity maxima of image that corresponds to the positions of atomic columns. On the basis of this, a transverse averaging of the shifts of each column species with respect to the cubic structure was plotted as a function of the vertical distance of Fig. 6a; the mean error is <5.6 pm for heavy Pb and Zr/O1/O2 columns and <10.6 pm for light oxygen columns with a confidence level of 95%. Second, for picometer precision measurements, the residual objective-lens aberrations and unavoidable tilting of the crystallographic zone axis away from the incident electron beam were examined in the image simulation to remove the deviation of the positions of contrast maxima in the image from the real atomic positions. For this, a procedure of iterative comparison of the calculated images with the experimental image was carried out through adjusting the image parameters on the basis of a perfect domain region in the experimental image until the best fit between the calculated and the experimental images was obtained. The used imaging parameters for the calculated image with the best fit were thus determined as the parameters used in the experiment. Afterwards, a structural model containing the APB was constructed for the image simulations based on the determined imaging conditions. An additional procedure of iterative comparison of the calculated images with the experimental image was carried out through adjusting the interfacial widths and the atomic positions near the boundary until the experimental image was best fitted by the calculated one. The structure model leading to the calculated image with the best fit to the experimental one provides the real atom positions at the APB area. The filtered experimental image and the simulated image with excellent match are shown in Fig. 6a,b.

**Figure 7:**
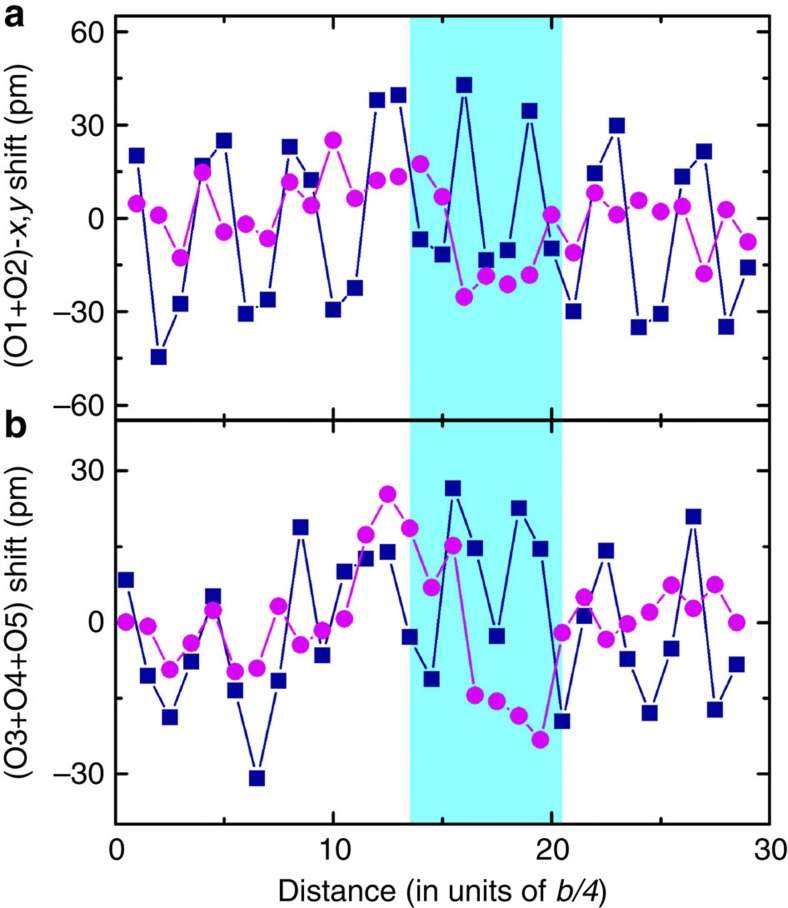
Atomic shifts of oxygen. Atomic shifts of (**a**) (O1+O2) atoms and (**b**) (O3+O4+O5) atoms derived from the final structure model along *x* (blue square) and *y* (pink circle) direction, respectively.

In the orthorhombic unit cell of PZ, oxygen atoms have five different crystallographic sites and lead atoms have two (Fig. 6c). Since the Pb1 (yellow) and Pb2 (light-blue below the yellow) overlap along the viewing direction and so do the O1, Zr and O2 atoms, the indistinguishable Pb1/Pb2 and O1/Zr/O2 atomic columns are moved as rigid entities in our image simulation. For clarity sake, the atomic position difference between Pb1 and Pb2 is not mentioned in the main text, and Fig. 3c presents the average atomic shifts of Pb1 and Pb2 derived from the final structural model. The displacements of overlapped O1 and O2 are summed up and shown in Fig. 7a. Ideally, there are no displacements for O4 and O5 atoms in the perfect domain region. Considering the antiphase rotation of the corner-linked octahedra, the displacements of O3, O4 and O5 atoms, which are directly observed in the experimental image, are also summed up to show the rotation behaviour of the octahedral (Fig. 7b). Note that in Fig. 7a,b, the displacement sums along *x*/*y* direction ([100]/[010]) are enhanced/reduced because of the same/opposite displacement directions of (O1 and O2)/O3 atoms, as clearly seen in Fig. 6c. In addition to the displacement values, re-arrangement of the oxygen atomic positions near the APB area reveals breaking of the antiphase-correlated rotation of the octahedra. The spontaneous polarization was calculated using the following formula:


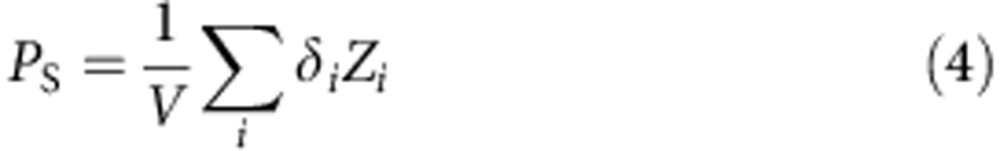


where *V*, *δ*_*i*_ and *Z*_*i*_ are the volume of the orthorhombic unit cell, displacement of atom *i* along the *x* or *y* direction from atomic positions of the centrosymmetric cubic perovskite structure and the effective charge of atom *i*. According to first-principles density-functional calculations on orthorhombic PbZrO_3_, the effective charges used in our calculation are *Z*_Pb_=3.9, *Z*_Zr_=6.0, *Z*_O1,O2_=−2.5, *Z*_O3,O4,O5_=−3.7 (see Supplementary Note 2). The spontaneous polarity for a sliding orthorhombic cell was calculated by atom displacements with respect to the cubic phase multiplied by the Born charges. By the ‘sliding’ cell we mean the orthorhombic cell of 40 atoms shifted each time along the [010] axis by *b*/4 (Fig. 6d).

### Modelling of local ferroelectricity in APBs in PZ

To perform first-principles calculations, we used Plane-Wave Self-Consistent Field programme, which is a part of the Quantum Espresso package^22^ performing zero Kelvin Density Functional Theory full relaxation calculations. The calculations were performed within the Generalized-Gradient Approximation with the Perdew–Burke–Ernzerhof exchange-correlation functional^23^ using ultrasoft pseudopotentials by Vanderbilt^24^. The kinetic energy cutoff for wavefunctions was 60 Ry (816 eV). The convergence threshold on forces for ionic minimization was chosen to be equal to 10^−3^ a.u. (21 meV  A^−1^).

## Author contributions

A.K.T. conceived the concept of the paper. N.S., A.K.T. and C.-L.J. initiated the experimental work. K.R. characterized PZ crystals electrically and optically. X.-K.W. carried out the electron microscopy experiments, sample treatment, image simulation and data analysis under the supervision of C.-L.J. A.K. performed *ab initio* calculations. X.-K.W. and A.K.T. wrote the manuscript with the help of C.-L.J., A.K. and N.S. All authors discussed the results.

## Additional information

**How to cite this article:** Wei, X.-K. *et al.* Ferroelectric translational antiphase boundaries in nonpolar materials. *Nat. Commun.* 5:3031 doi: 10.1038/ncomms4031 (2014).

## Supplementary Material

Supplementary Information Supplementary Figures 1-2, Supplementary Notes 1-2 and Supplementary References
