# Supplementary material for: Ferroelectric translational antiphase boundaries in nonpolar materials
Source: Nat Commun. 2014 Jan 8;5:3031. doi: 10.1038/ncomms4031 (PMC3941019; doi:10.1038/ncomms4031)
Supplement: Supplementary Information — Supplementary Figures 1-2, Supplementary Notes 1-2 and Supplementary References [file ncomms4031-s1.pdf]

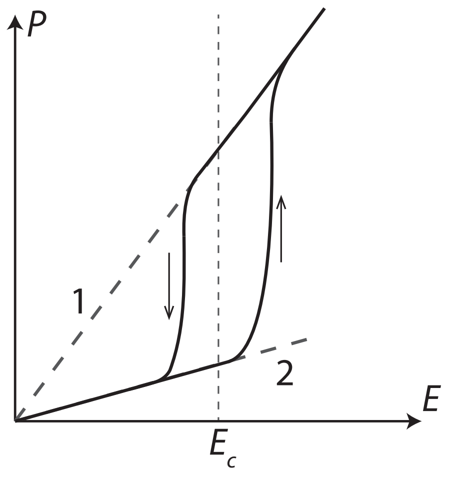


Supplementary Figure 1. **Double hysteresis loops from the two-instability model for antiferroelectricity.** Schematic $P-E$ dependences: obtained from supplementary relationship (9) - dashed line 1, obtained from the set of supplementary equations (6) and (5) - dashed line 2, the total $P-E$ dependence taking into account the phase coexistence in the vicinity the first-order phase transition - solid blue curve.

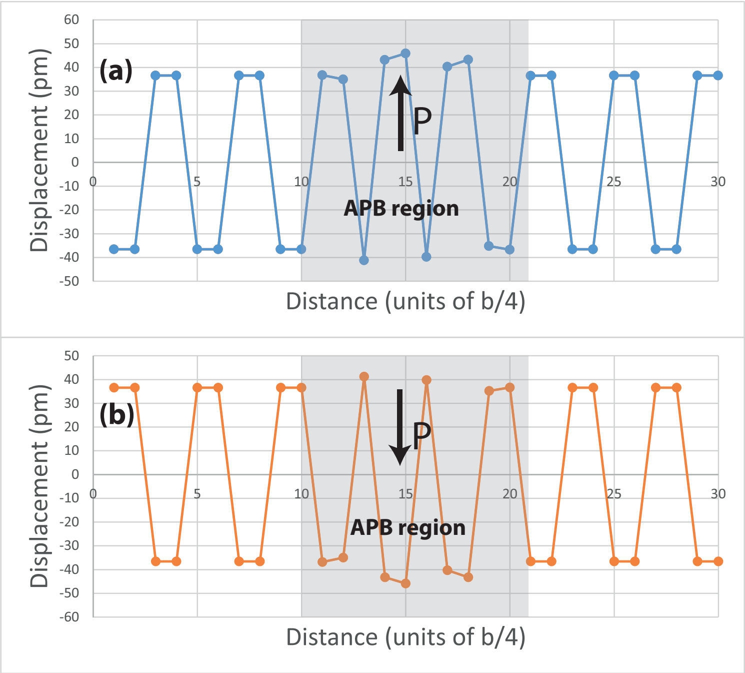


Supplementary Figure 2. **Pb atom displacements from cubic positions along [100] direction in the APB region.** There are 2 equivalent configurations (a) and (b) corresponding to the same energy confirming the bistability of the structure.

## SUPPLEMENTARY NOTE 1. DEFINITION OF ANTIFERROELECTRICITY AND TWO-INSTABILITY MODEL

**Comment on definition of antiferroelectricity and the nature of the structural order parameter which controls the antiferroelectric phase transition**

The definition of antiferroelectric phase transition given in the main text (a transition between two non-polar phases accompanied with a strong dielectric anomaly at its high-temperature side) is actually its macroscopic definition. It is such definition that enables experimental identification of this phenomenon. This definition perfectly suits the message of our paper. However, typically, antiferroelectric phase transitions are accompanied with unit cell multiplication and staggered order parameter. Remarkably, these features themselves will not ensure that the material is antiferroelectric. A good illustration for this point is the antiferrodistorsive transition in strontium titanate which exhibits both the unit cell multiplication and staggered order parameter but which is not antiferroelectric. One may reproduce antiferroelectric behaviour using Kittel model[25] explicitly incorporating the staggered order parameter. However, this is done at the expense of a strong assumption. It is important to note that there is no analogy between the set of the properties of antiferromagnets and that of antiferroelectrics. So that the arguments based on such analogy are misleading.

The two-instability model[12,15] provides the simplest description of the key features of antiferroelectrics. An essential feature of this phenomenological model is that no structural or symmetry limitations are imposed on this order parameter, except that it should not carry dipole electric moment. This means that under the elements of the crystalline symmetry of the material, the order parameter should not transform like a polar vector. This ensures that the low symmetry phase is non-polar (as it should be in an antiferroelectric). In parallel, this order parameter can participate in the bi-quadratic repulsive coupling with polarization, providing the suppression of the ferroelectric instability at the transition. In principle, there is no reason to believe that a antiferroelectric phase transition without unit cell multiplication is impossible.

**Explanation of the appearance of the double polarization field (P-E) hysteresis loop using the two-instability model for antiferroelectricity**

In the main text, the characteristic dielectric anomaly accompanying an antiferroelectric phase transition has been explained using the two-instability model. Another characteristic feature of such transitions is the observation of double polarization-field ($P - E$) hysteresis loops. Below we demonstrate how the double hysteresis loops can be explained using the same model. First, we specify a Landau expansion (Eq.(1) from the main text) so that it explicitly describes a first order phase transition with respect to the order parameter $\xi$ at $T_\mathrm{A}$:

$$F(P,\xi) = \frac{1}{2}A(T - T_0)P^2 + \frac{1}{2}\delta_\mathrm{P}P^2\xi^2 + \frac{1}{2}\alpha(T - T_0^\mathrm{str})\xi^2 + \frac{1}{4}\beta\xi^4 + \frac{1}{6}\gamma\xi^6 \tag{1}$$

where $\beta < 0$, $\gamma > 0$, and $T_0^\mathrm{str}$ is the extrapolated instability temperature for the antiferroelectric phase transition. From the theory of first order phase transitions[18] we derive the equation of state for $\xi$

$$\alpha(T - T_0^\mathrm{str}) + \beta\xi^2 + \gamma\xi^4 = 0 \tag{2}$$

and

$$\xi_0^2 = -\frac{3\beta}{4\gamma} \tag{3}$$

$$\alpha(T_\mathrm{A} - T_0^\mathrm{str}) = \frac{3\beta^2}{16\gamma} \tag{4}$$

where $\xi_0$ is the spontaneous value of the order parameter $\xi$ at the transition temperature (i.e. at $T = T_\mathrm{A}$). The application of a dc electric field $E$ induces a polarization $P$, modifying the equation of state (supplementary equation (2)) to the following:

$$\delta_\mathrm{P}P^2 + \alpha(T - T_0^\mathrm{str}) + \beta\xi^2 + \gamma\xi^4 = 0 \tag{5}$$

where $P$ satisfies the equation of state $\partial F/\partial P = E$ which can be rewritten as

$$[A(T - T_0) + \delta_\mathrm{P}\xi^2]P = E. \tag{6}$$

Supplementary equation (5) implies a field-induced lowering of the transition temperature (owing to the positive sign of $\delta_\mathrm{P}$). This means that at a temperature $T$ in the antiferroelectric phase, the applications of a large enough electric field could possibly shift the transition temperature down to this temperature. Thus, at a fixed temperature, one can speak about a field-induced first-order phase transition with respect to the order parameter $\xi$. In general,

this phase transition is accompanied with a sudden change in the dielectric permittivity. Since this jump occurs at a finite value of the electric field, when considered along with the first order phase transition described above, it implies double hysteresis loops on the $P-E$ curve.

Alternatively, a more detailed description of this effect is as follows. Combining supplementary equations (4) and (5) one finds the value of the field-induced polarization, $P_{\mathrm{C}}$, corresponding to field-induced shift of the transition temperature $T_{\mathrm{A}}$ down to temperature $T$:

$$P_{\mathrm{C}}=\sqrt{\frac{\alpha(T_{\mathrm{A}}-T)}{\delta_{\mathrm{P}}}}. \tag{7}$$

The critical field for the field-induced phase transition, $E_{\mathrm{C}}$, following from supplementary equations (6), (7), and (3), is given by

$$E_{\mathrm{C}}=\sqrt{\frac{\alpha(T_{\mathrm{A}}-T)}{\delta_{\mathrm{P}}}}\left[A(T-T_0)-\delta_{\mathrm{P}}\frac{3\beta}{4\gamma}\right]. \tag{8}$$

Thus, at $T<T_{\mathrm{A}}$ in the antiferroelectric phase, the state with $\xi\neq 0$ is energetically favorable for $E<E_{\mathrm{C}}$. Conversely, the state with $\xi=0$ is favorable for $E>E_{\mathrm{C}}$. Therefore, neglecting a possible coexistence of phases in the vicinity the first-order phase transition, for $E>E_{\mathrm{C}}$ the dielectric response of the system is controlled by the relationship

$$A(T-T_0)P=E \tag{9}$$

while for $E<E_{\mathrm{C}}$ it is controlled by the set of supplementary equations (6) and (5). Using these equations, if $P$ is plotted as a function of $E$ while taking into account the phase coexistence around $E_{\mathrm{C}}$, one finds that this dependence clearly shows "antiferroelectric" hysteresis loops as shown schematically in Supplementary Figure 1.

The above discussion elucidates the origin of the double hysteresis loops observed below the antiferroelectric phase transition. These loops correspond to the field-induced first order phase transition between the low-symmetry and high-symmetry phases with respect to the structural order parameter $\xi$. Hence, we believe there is no ground to use the term "field induced ferroelectric state" for the state of the system at $E>E_{\mathrm{C}}$.

One should mention that formally, the model discussed will still exhibit the double hysteresis loops if the ferroelectric instability is absent[26], just a structural ("non-ferroelectric") first order phase is needed. However, in reality, without the ferroelectric instability providing

the enhanced values of dielectric susceptibility, the critical field for the field-induced phase transition $E_{\mathrm{C}}$ will be unrealistically high.

**The appearance of ferroelectric phase when the antiferroelectric phase is modified by dopants or by hydrostatic pressure**

The two-instability model can also reproduce a possibility to turn an antiferroelectric into a ferroelectric by small chemical modification or the application of hydrostatic pressure. Indeed, in view of small deference between temperatures $T_0$ and $T_{\mathrm{A}}$ , which is the key element of the model, the aforementioned actions may swop the relative positions of these temperatures. In this case, on cooling, the ferroelectric phase transition will takes the place first and the material becomes a ferroelectric. Such scenario corroborates with the appearance of ferroelectricity once PZ is slightly doped with Ti[27].

## SUPPLEMENTARY NOTE 2: MODELING OF LOCAL FERROELECTRICITY IN ANTIPHASE BOUNDARIES IN LEAD ZIRCONATE

As a starting point for the calculations we obtained a relaxed PZ orthorhombic cell of 40 atoms with $a = 6.056$ Å, $b = 11.954$ Å and $c = 8.334$ Å (experimental values are $a = 5.884$ Å, $b = 11.787$ Å, $c = 8.231$ Å[28]) exhibiting all the specific features of the structure, such as 8 times multiplication of the cubic unit cell, antiparallel Pb atom displacements, and O octahedron rotations. This result is consistent with first principles results obtained by Waghmare and Rabe[29]. Further, we constructed a 220 atoms super cell (5.5 orthorhombic cells) simulating a $\pi$ phase shift in APB. The two end cells of the supercell had a fixed orthorhombic structure corresponding to that of the PZ inside the adjacent domains and the inner 3.5 orthorhombic cells were relaxed. Spontaneous polarization for a "sliding" cell was calculated by atom displacements with respect to the cubic phase multiplied by Born charges calculated with QE ($Z_{\mathrm{Pb}} = 3.889, Z_{\mathrm{Zr}} = 5.996, Z_{\mathrm{O1,O2}} = -2.464, Z_{\mathrm{O3,O4,O5}} = -3.718$).

We would like to underline that we calculate polarization using Born charges (and not Berry phase approach) on purpose. The reason for this is that we show our *ab initio* results in comparison with the experiment, namely we compare the atomic behaviour in the APB region using experimental data and first principles calculations. The "experimental"

polarization was calculated by atomic displacements and Born charges, this way we believe that it is worth calculating the "*ab intio*" polarization using the same approach for a clearer comparison with the experiment.

We estimated the surface formation energy of the $\pi$-wall considered above. We compared the energies of the super cell (5.5 orthorhombic cells) simulating the APB region and of the 5.5 cells of the bulk PZ. The value of the surface formation energy was found to be about 190 mJ $m^{-2}$.

The width of the APB region was found to be about 2 orthorhombic cells. The calculations showed the presence of local polarity in APB. Moreover, the energy of the two configurations of atom displacements shown schematically for lead atoms in Supplementary Figure 2 was found to be equivalent. Thus the structure was found to be bistable. Hence the ab initio calculations showed both, the presence of local polarity, and the possibility of polarization switching, supporting the ferroelectric nature of APB.

## SUPPLEMENTARY REFERENCES


[25] Kittel, C. Theory of antiferroelectric crystals. *Phys. Rev.* **82**, 729–732 (1951).

[26] Strukov, B. A. & Levanyuk, A. P. *Ferroelectric Phenomena in Crystals* (Springer, Berlin, 1998).

[27] Jaffe, B., Cook, W. J. & Jaffe, J. *Piesoelectric Ceramics* (Academic Press, London, 1971).

[28] Corker, D. L., Glazer, A. M., Dec, J., Roleder, K. & Whatmore, R. W. A re-investigation of the crystal structure of the perovskite $PbZrO_3$ by x-ray and neutron diffraction. *Acta Cryst.* **B53**, 135–142 (1997).

[29] Waghmare, U. V. & Rabe, K. M. Lattice instabilities, anharmonicity and phase transitions in $PbZrO_3$ from first principles. *Ferroelectrics* **194**, 135–147 (1997).
